# Don't You Just Hate Peer Review?

**DOI:** 10.5812/traumamon.10396

**Published:** 2013-05-26

**Authors:** Mohammad Hosein Kalantar Motamedi

**Affiliations:** 1 Trauma Research Center, Baqiyatallah University of Medical Sciences, Tehran, IR Iran

**Keywords:** Peer Review, Manuscripts, Medical, Periodicals

**Manuscript Submission.** Don't you just hate it when your submission to the journal office has to be sent-out for peer review? Don't you hate awaiting comments of the peer reviewers on your manuscript not knowing when the replies of the reviewers are going to come in? After several weeks or months of anticipation, when the replies from the reviewers are finally in, don't you just hate it when their comments and their recommended changes happen to contradict one another and you don’t know which reviewer’s comments to implement and end up having to write a rebuttal? 

**Manuscript Revision.** Don't you just hate the laborious and arduous task of revising and re-submitting a revised manuscript along with the point-by-point replies-to-reviewers back to the journal office for the editor to request reassessment from the reviewers who must check and see if the revisions made in the manuscript are adequate and if they are satisfied with the changes made? Don’t you hate having to "wait-it-out" again, not knowing if your revised manuscript is to be accepted, rejected or returned for further revisions? 

**Manuscript Re-revision.** After several re-revisions when your manuscript is deemed not acceptable for publication and is ultimately rejected, doesn’t that make you just hate peer review? Well, actually it shouldn't; because despite all this, the peer review process is the “gold standard” of accredited medical journals worldwide. Without peer review a medical journal is just another magazine or a newspaper that presents various viewpoints without verifiable documentation or approval of specialists in the same scientific field of study. 

**Peer review.** Peer review is the evaluation of creative work or performance by other individuals in the same profession with the intent to maintain or enhance the quality of the work or performance in that field (1). Peer review means that actions or statements of an individual are looked-at again (reviewed) by someone of similar competence in that subject or activity - a peer (2). 

**Figure 1.**
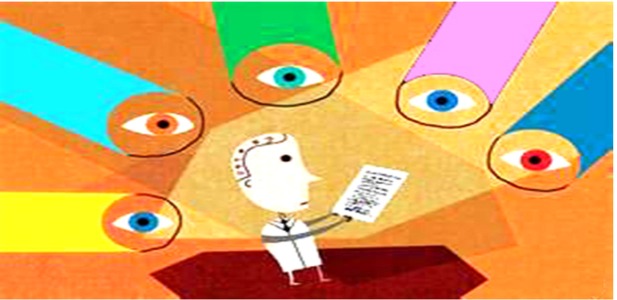
Metaphor of a Medical Paper Undergoing Peer Review

More formally, peer review constitutes a process of self-regulation by a professional or a process of evaluation involving qualified individuals within the relevant field. Therefore, a medical manuscript must be assessed by a medical professional of the same specialty; because, only a peer can judge if a submission is original, reliable and authentic and whether or not it merits publication. Peer review methods are employed to maintain standards, improve quality and provide credibility. In academia peer review is often used to determine an academic paper's suitability for publication (Figure 1) ( 2 ). Peer review constitutes the basis for credibility and authenticity of medical journals and the reliability of the research methodology presented therein; thus, although the peer review process is pain-staking, time-taking, tedious and frustrating, the fact of the matter is that without it, not much of what is stated can be substantiated.
